# IgM-MGUS and associated membranoproliferative glomerulonephritis during IVIG administration

**DOI:** 10.1007/s00277-020-04046-x

**Published:** 2020-05-04

**Authors:** Johannes Ruhe, Hermann-Josef Gröne, Mandy Schlosser, Claus Kroegel, Gunter Wolf, Martin Busch

**Affiliations:** 1 Department of Internal Medicine III, Jena University Hospital, Am Klinikum 1, 07747 Jena, Germany; 2 Department of Cellular and Molecular Pathology, German Cancer Research Center (DKFZ), Heidelberg, Germany; 3 Department of Internal Medicine I, Jena University Hospital, Jena, Germany

**Fig. 1:**
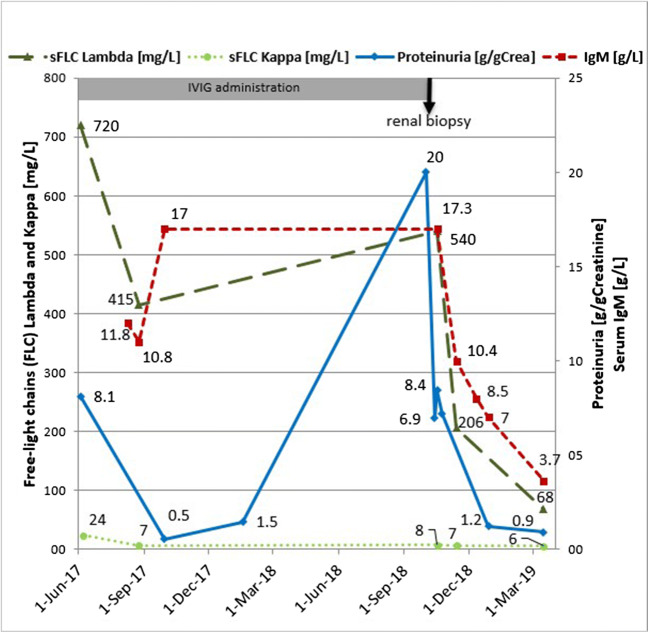
Proteinuria, serum IgM, and serum lambda free-chains (sFLC) lambda and kappa before and after cessation of intravenous immunoglobulins (IVIG) as measured after diagnosed IgM-monoclonal gammopathy type lambda

Dear Editor,

A 61-year-old woman with IgM-monoclonal gammopathy of unknown significance (MGUS) type lambda was transferred to our department in October 2018 showing a severe nephrotic syndrome. Renal function was not impaired. Due to a common variable immunodeficiency (CVID) syndrome with hypogammaglobulinemia (IgA and IgG), the patient received polyvalent human IVIGs in a dose of 20 g every 3 to 4 weeks since 2014. The immunoglobulin preparation used (Privigen®, CSL Behring GmbH, Germany) contains IgG1 67.8%, IgG2 28.7%, IgG3 2.3%, IgG4 1.2% and IgA at a maximum of 25 μg/mL, 250 mmol/L L-proline and water [1]. The course of free light chains (FLC), serum IgM and proteinuria since initial diagnosis of MGUS is shown in Fig. 1.

Current findings showed reduced complement factors (C3: 0.41 g/L [NR: 0.78–1.51 g/L]; C4: 0.11 g/L [NR: 0.12–0.33 g/L]) suggesting an immunocomplex-mediated disease. ANA, ds-DNA, cryoglobulins, p- and c-ANCA-antibodies were normal. Serologies for HIV, hepatitis B and C were negative. Ultrasound diagnostics and chest x-ray showed pulmonary and hepatic congestion. Echocardiography was normal. Diuretic treatment was initiated, leading to adequate weight loss. Renal biopsy was performed. Findings were compatible with a membranoproliferative glomerulonephritis (MPGN) of the immune complex type with deposits of IgM matching the serological findings of the paraproteinemia [2]. Repeatedly performed bone marrow biopsy excluded again Waldenström macroglobulinemia, myeloma or other hematological malignancy, a CT-scan ruled out osteolysis.

The preexisting high-risk IgM-MGUS was updated to that of IgM-MGRS [3–5]. Assuming a potential effect of the IVIGs on the development of MPGN, they were discontinued. In fact, serum levels of IgM spontaneously dropped rapidly and nearly normalized within 23 weeks (17.3 g/L to 3.65 g/L; NR: 0.46–2.92 g/L) as well as serum free light chains (sFLC) lambda (540 mg/L to 68 mg/L; NR: < 26.3 mg/L) and proteinuria (20 g/gCrea to 0.9 g/gCrea; NR: < 0.1 g/gCrea) (Fig. 1). In parallel, IgG levels decreased as expected after cessation of the IVIG therapy. Therefore, IVIG therapy was reintroduced in reduced dosage (10 g) and prolonged time interval (4 weeks). Following this regime, the patient had no episode of infection whilst having a further decreasing proteinuria (0.49 g/gCrea in July 2019).

Surprisingly, not only nephrological but also hematological results improved enormously to a point that neither criteria for MGRS nor even MGUS were fulfilled [6].

The parallel decrease of IgM, sFLC-lambda and proteinuria suggests a potential role for IVIGs in the pathogenesis of MGUS and the resulting IgM-positive MPGN in this patient. A direct effect of IVIGs is unlikely as the IVIG preparation used was free of IgM. However, IVIGs are known to also contain natural auto-antibodies including IgM type auto-antibodies [7]. In addition, idiotypic receptors on plasma cells secreting IgM may be activated by anti-idiotypic antibodies in the IVIG preparation resulting in stimulated IgM synthesis [8].

We speculate that IVIGs induced an acute immune response leading to monoclonal IgM production followed by its renal deposition causing glomerular immune reactions. To our knowledge, this is the first case describing a reversible high-risk MGUS and MGUS-associated immune complex-mediated MPGN provoked by the administration of IVIGs. Yet, the precise immune mechanisms underlying this observation remain unclear.
